# Sum-Frequency Scattering Spectroscopy Reveals the Charging Mechanism and Surface Structure of hBN Nanoflakes in Solution

**DOI:** 10.1021/acsnano.5c03589

**Published:** 2025-07-03

**Authors:** Benjamin Rehl, Nathan Ronceray, Li Zhang, Aleksandra Radenovic, Sylvie Roke

**Affiliations:** † Laboratory for Fundamental BioPhotonics, Institute of Bioengineering (IBI), School of Engineering (STI), École Polytechnique Fédérale de Lausanne (EPFL), CH-1015 Lausanne, Switzerland; ‡ Laboratory of Nanoscale Biology (LBEN), Institute of Bioengineering, School of Engineering, Swiss Federal Institute of Technology Lausanne (EPFL), 1015 Lausanne, Switzerland; § Institute of Materials Science and Engineering (IMX), School of Engineering (STI), École Polytechnique Fédérale de Lausanne (EPFL), CH-1015 Lausanne, Switzerland; ∥ Lausanne Centre for Ultrafast Science, École Polytechnique Fédérale de Lausanne (EPFL), CH-1015 Lausanne, Switzerland; ⊥ NCCR Bio-Inspired Materials, 37580 École Polytechnique Fédérale de Lausanne, 1015 Lausanne, Switzerland

**Keywords:** liquid exfoliated 2D materials, liquid inks, hexagonal boron nitride, vibrational sum-frequency scattering spectroscopy, charge transfer, ζ-potential

## Abstract

A molecular understanding of the interactions between two-dimensional (2D) layered materials and liquids is crucial for nanofluidics, catalysis, and solution-based 2D material processing. Among 2D materials, hexagonal boron nitride (hBN) has a number of outstanding properties, but its interactions with liquids remain poorly characterized. Here, we investigate the interfacial structure of few-layer hBN nanoflakes suspensions in ethanol and ethanol–water mixtures. Electrophoretic light scattering suggests that the nanoflakes are effectively positively charged in ethanol and negatively charged in an ethanol–water mixture. Vibrational sum-frequency scattering spectroscopy reveals the surface structural changes underlying this charge reversal. Signatures of charge transfer of opposite direction are detected on both the flake lattice and in the liquid. The different (partial) charge distributions in ethanol and water explain the apparent charge reversal.

## Graphical abstract

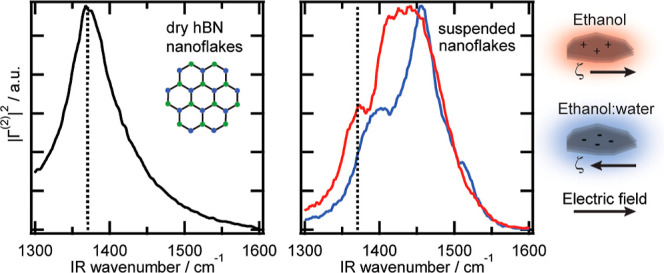

## Introduction

Liquid-exfoliated two-dimensional (2D) materials have gained significant attention for their use in liquid inks. 2D materials exhibit unique properties compared to their bulk counterparts, for example, the enhanced conductivity of graphene relative to graphite, making them highly promising for applications in printable optoelectronics, photonics, sensors, and batteries.^1^ Compared to alternative preparation methods, including chemical vapor deposition and mechanical exfoliation, liquid exfoliation offers advantages such as high yield, tunable flake size and distribution, and lower cost, which are key factors for enabling the large scale deployment of these technologies.^2^ Central to the liquid exfoliation process is the complex and diverse interfacial behavior of 2D materials with solvents. However, despite its significance, the nature of these interactions between nanoflakes and solvents on the molecular scale remains poorly understood.

Among the vast array of over 3000 materials that can exist as 2D materials,^3^ hexagonal boron nitride (hBN) nanoflakes are particularly promising for printable transistors^4^ and dielectric inks^5^ owing to its large bandgap, corrosion resistance,^6^ high thermal conductivity,^7^ and ambipolar behavior showing strong affinities for both water and organic solvents. This dual affinity is reflected in several key interfacial phenomena: water flow through hBN nanotubes can be explained by no-slip flow,^8,9^ alkanes under frozen and ultrahigh vacuum conditions order at the surface of hBN differently than at the surface of graphite,^10^ and organic solvents activate fluorescent emission from pristine hBN crystals which was interpreted to reveal interfacial molecular random walks.^11–13^ These studies highlight the importance of the flake-solvent interface, which has significant implications for solution processing and flake suspension stability. For example, specific solvent interactions with hBN crystals have been leveraged to develop liquid-phase exfoliation methods,^14–18^ including a cosolvent approach that combines water with polar organic solvents.^19^ Although these empirical strategies exploit hBN’s competing affinities to enhance exfoliation efficiency, the underlying molecular mechanisms are unknown. This deficient understanding arises from a lack of characterization methods^4^ that can relate the macroscopic properties to molecular level structure. The molecular view, in particular, of the interactions between hBN nanoflakes and solvent molecules has been underreported.

To potentially remediate this lack of knowledge, vibrational sum-frequency generation (SFG) can be used. SFG probes the second-order nonlinear susceptibility, **χ** ^(2)^, of interfaces, which contains molecular level information about the interface, typically up to ∼1 nm deep. Under the electric-dipole approximation, SFG requires an anisotropic arrangement of molecules,^20^ and therefore this technique is uniquely suited to probe solid–liquid interfaces, as they are inherently noncentrosymmetric in the direction of the surface normal. Briefly, when illuminating a sample with visible (VIS) and infrared (IR) laser pulses, the anisotropic arrangement of molecules, represented by the surface susceptibility **χ_s_** ^(2)^ of the interface is responsible for the generation of a second-order polarization, a charge oscillation occurring at a frequency ω_SFG_ = ω_IR_ + ω_VIS,_ which leads to the emission of light at the sum frequency. In vibrational SFG, the infrared frequency ω_IR_ is tuned to match vibrational frequencies of the material under study, which results in a resonant enhancement of the SFG light and therefore SFG is sensitive to the interfacial chemical environment (e.g., hydrogen (H) bonding, the presence of functional groups, etc.). Modes that are SFG active are both IR and Raman active. Previously, SFG was used to study interfacial water on graphene electrodes deposited on CaF_2_ substrates.^21–23^ Graphene, although similar in configuration to hBN, is centrosymmetric (point group *D*_6h_), and its pristine lattice vibrations cannot generate any sum-frequency light, and thus SFG from water at graphene electrodes should originate entirely from interfacial water. However, the results were found to be significantly influenced by the substrate used to support graphene.^23^

Vibrational sum-frequency scattering (SFS) spectroscopy is a substrate-free variant of SFG, which probes the interfaces of nano-objects that are freely floating in suspension. Therefore, this technique circumvents substrate-induced effects, and provides information about the neat flake-solvent interactions. Instead of determining the **χ_s_** ^(2)^ tensor of a planar extended interface, as is done in reflection SFG studies, SFS measures the effective particle susceptibility **Γ** ^(2)^(ω_IR_, ω_VIS_),^24^ which contains information about **χ_s_** ^(2)^ as well as the size and the shape of the flake as detected in a certain geometry. SFS achieves a very high sensitivity enabling measurements of the interfacial structure of nano-objects such as water droplets,^25^ oil droplets,^26^ and liposomes.^27–29^ Previously, noncentrosymmetric 2D crystals in suspension were investigated with second-harmonic scattering (SHS). In these experiments, suspended 2D transition metal dichalcogenide crystal flakes^30^ were characterized in terms of their bulk lattice response, which is generally much stronger than the interfacial response. The interfacial molecular structure of such flakes and flake-solvent interactions have, so far, not been reported.

Herein, we combine electrophoretic light scattering with vibrational sum-frequency scattering spectroscopy to develop a molecular understanding of hBN nanoflake/solvent interactions. From a macroscopic view, the ζ-potential of hBN nanoflakes dispersed in ethanol and an ethanol–water mixture are of opposite sign. This observation hints at flake-solvent interactions that are responsible for charge inversion. Comparing the sum-frequency spectra of dry, drop-casted hBN flakes to suspended ones, a notable blueshift is observed in the B–N stretch vibration when the flakes are suspended in liquid. This shift is stronger when water is present. These vibrational shifts further indicate flake-solvent interactions are occurring. The presence of C–H stretches and O–H/O–D stretches validates the presence of solvent molecules at the nanoflake surface. From an analysis of these modes, we determine that the ethanol molecules prefer a more parallel orientation with the flake surface on average. Significant improper H-bonding between the B–N groups of the flake and O–D groups of the water is occurring. Therefore, we conclude that the mechanism for the charging behavior of the hBN nanoflakes originates from charge transfer between the flake and solvent molecules. In neat ethanol, this charge transfer yields positively charged flake-solvent ensembles as seen by an external electrostatic field, since the partial positive charges are not mobile. When water is present, positive charge can delocalize throughout the water H-bond network. This decoupling between the negative and positive charges inverts the sign of the flake mobility.

## Results and Discussion

### Electrophoretic Mobility of hBN Nanoflake Suspensions

The solvent-dependent behavior of hBN was measured with electrophoretic light scattering (ELS) (Figure 1a). Mobility values were converted into ζ-potentials using the Henry function.^31^ A surfactant-free commercial suspension of few-layer pristine hBN (Graphene Supermarket) in 55 v/v % ethanol/water was used. It contains ∼5 mg/L of hBN nanoflakes with thicknesses of 1–5 layers,^32^ each approximately 3.4 °A thick, with a lateral size ranging from 50 to 200 nm.^33^ We also measured the ζ-potential of these flakes redispersed in absolute ethanol. Notably, a charge reversal between the two solvent systems was observed, with a ζ-potential of +28.4 mV and −41.5 mV for flakes dispersed in ethanol and ethanol/water, respectively. The size distribution measured by dynamic light scattering were similar between the two suspensions with sizes in agreement to previous characterization through electron and atomic force microscopies^32–34^ (Figure 1b), indicating no influence on the ζ-potential due to the particle size. However, the flakes suspended in ethanol exhibited a slow increase in average size over time, indicating an aggregation process arising from a decrease in stability in the absence of water (Figure S1). With no additives in the system, the reversal in electrophoretic mobility indicates that interfacial effects are playing a key role in the charging behavior. To further understand these interfacial effects, a molecular level view of the relevant interfacial species is required.

**Figure 1:**
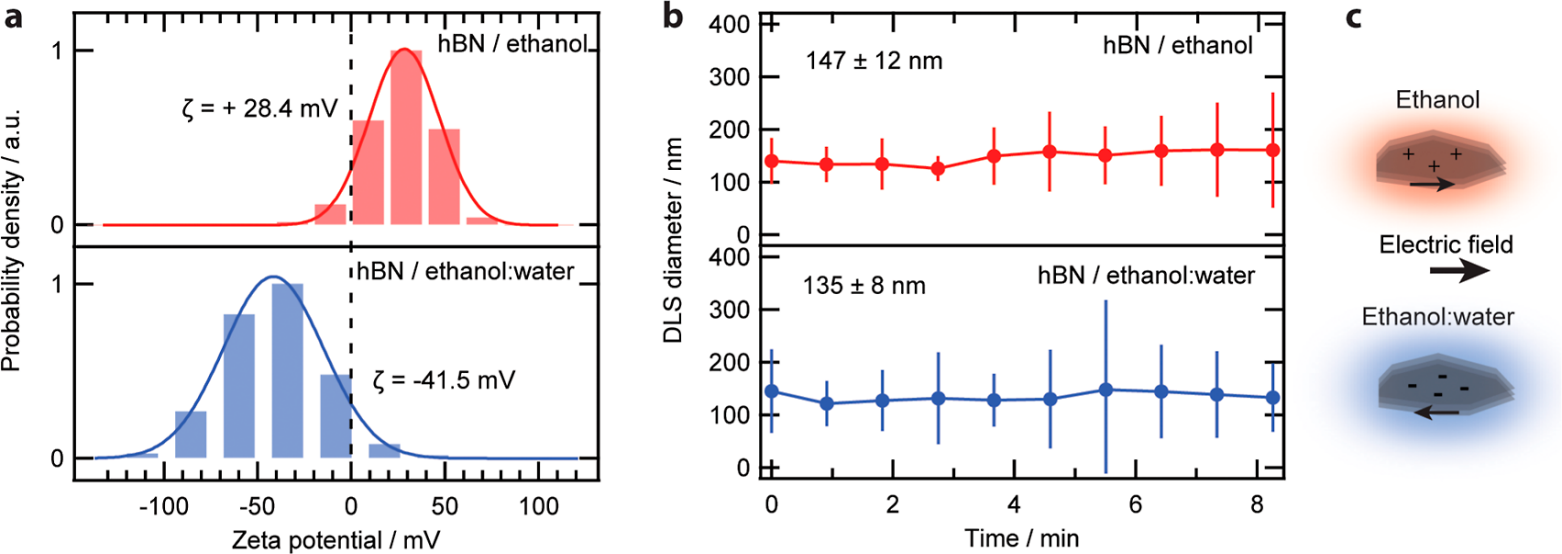
Characterization of the hBN nanoflake suspensions. (a) ζ-potential distribution obtained from electrophoretic light scattering, showing a change in sign between the two suspensions. (b) Mean flake size of the flake suspension in EtOH (top) and in EtOH/H_2_O (bottom) measured through dynamic light scattering (DLS) as obtained by fitting the DLS data to a log–normal distribution. The DLS size corresponds to the distribution peak and error bars denote the linear scale standard deviation of the distribution. (c) Sketch of the reversal of electrophoretic mobility.

### Vibrational Spectra of hBN Nanoflakes

We measured vibrational SFS spectra of hBN nanoflakes dispersed in ethanol and 55 v/v % ethanol/water in the spectral region of the B–N stretch modes around 1370 cm^–1^ (Figure 2b). To avoid interference from the C–H bending modes which vibrate in the same spectral window (see Figure S2), SFS spectra were recorded from flakes dispersed in deuterated solvents. As the C–D bend modes are shifted to lower frequencies compared to the C–H bend modes, no spectral interference occurs in this case. We observed strong sum-frequency intensity despite being close to the particle number and radius detection limit for spherical particles,^35^ likely owing to the increased surface area to volume ratio of nanoflakes, and because some of the modes arise from 2D crystal lattices. Figure 2b shows broad spectral lineshapes in the range from 1300 to 1550 cm^–1^ for hBN flakes dispersed in both solvent systems. These spectra exhibited multiple features and a shoulder at lower frequencies. However, the shoulder for flakes in ethanol was red-shifted relative to that of flakes in ethanol/water. To benchmark these B–N vibrations, attenuated total reflectance infrared (ATR-IR) spectroscopy, and confocal micro-Raman spectroscopy (μ-Raman) measurements were taken, as well as SFS recordings of dry, drop-casted hBN films (Figure 2c). In the IR and Raman spectra, we observed a peak centered at 1381 and 1366 cm^–1^, respectively, which in addition to the narrow Raman line width (∼10.5 cm^–1^), is indicative of highly crystalline hBN but may be slightly broadened by a moderate finite-size effect^36^ and the thickness variations of the film.^32^ In the SFS spectrum of the flake film, we observed a single peak around 1367 cm^–1^ that was asymmetric due to interference with the strong nonresonant background. By comparison to the IR and Raman spectra, we assign this mode in the SFS spectrum of dry flakes to the B–N stretch of the flake. Relative to the spectrum of dry flakes, the line shape as a whole is blue-shifted for flakes suspended in ethanol and ethanol–water. The presence of such a shift is indicative of interactions between the nanoflake and the solvent molecules.

**Figure 2:**
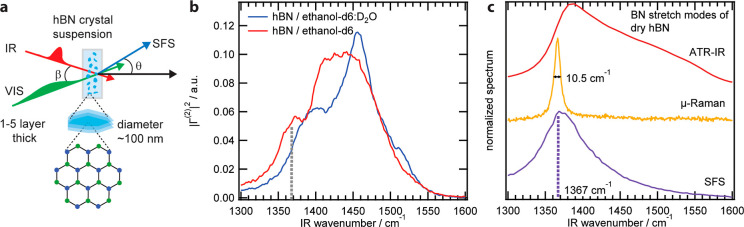
Vibrational spectra of hBN nanoflakes. (a) Sketch of an SFS measurement of the hBN suspension. (b) SFS spectra of hBN suspensions in the B–N stretching region for ethanol-*d*_6_ (red) and the ethanol-*d*_6_/D_2_O (blue) mixture. The dashed lines indicates the B–N stretch peak position of the dry flakes. (c) Vibrational spectra of the drop-cast nanoflakes through infrared spectroscopy (ATR-IR, red), Raman spectroscopy (μ-Raman, yellow) and SFS (purple).

### Probing Ethanol at the hBN Nanoflake Surface

Next, we measured the sum-frequency spectra of hBN flakes suspended in (protonated) ethanol and ethanol/water mixtures (the same solutions as in Figure 1) in the C–H stretching region to probe interfacial ethanol and the O–D stretching region to probe interfacial water. The C–H spectra are shown in Figure 3a, which were processed to remove the effects of IR absorption by the bulk solvent. The procedure is described in refs 37 and 38 . A vibrational SFG study at the air-ethanol interface by Wang and co-workers^39^ conducted using various forms of selective deuteration, concluded that interfacial ethanol exhibits three vibrational modes at 2875 cm^–1^, 2930 cm^–1^, and 2970 cm^–1^, which are assigned to the overlap of a strong methyl symmetric stretch (ss-CH_3_) and a weak methylene symmetric stretch (ss-CH_2_), a Fermi resonance of the ss-CH_3_ mode, and the overlap of a strong asymmetric methyl stretch (as-CH_3_) with a weak Fermi resonance of the ss-CH_2_ mode, respectively. Figure 3a shows that these identified C–H modes of ethanol are also present at the hBN nanoflake interface in pure ethanol (red) and a 55 vol % ethanol/H_2_O mixture, with the peak at ∼2970 cm^–1^ being the main contributor. In both cases the |**Γ** ^(2)^ *|* ^2^ response is much weaker than that of the B–N stretching region in Figure 2b. The weaker intensity compared to that of the B–N modes is due to the hBN lattice which does not have a center of inversion symmetry. This leads to coherent enhancement of the sum-frequency intensity.^20^ In contrast, while there are nonzero orientational correlations between the methyl and methylene groups of interfacial ethanol molecules there is far less constructive interference as in the case of the B–N modes.^40^ The combination of mode intensities in the pure ethanol (red) spectrum, with the as-CH_3_ mode being strong and the ss-CH_3_ mode being weaker provides some information about the average orientational distribution. This observed mode combination suggests that the CH_3_ groups are oriented such that the primary axis of the methyl group has a predominantly parallel orientation with respect to the surface plane. This type of spectral shape has been observed previously for alkane molecules at oil droplets dispersed in water.^41^ Comparing now the ethanol (red) and ethanol/water (blue) spectra, there is a marked difference in intensity, which could be either due to a change in the orientational distribution or to a difference in the number of ethanol molecules at the interface. There is also a broad background overlapping with the C–H modes. Both observations are potentially explained by the presence of water at the interface. If some of the ethanol was replaced by water, it would lead to a reduction in intensity of the C–H modes combined with the appearance of a weakly dispersive background, which one typically sees in the spectral region adjacent to the O–H stretch modes.^41^

**Figure 3:**
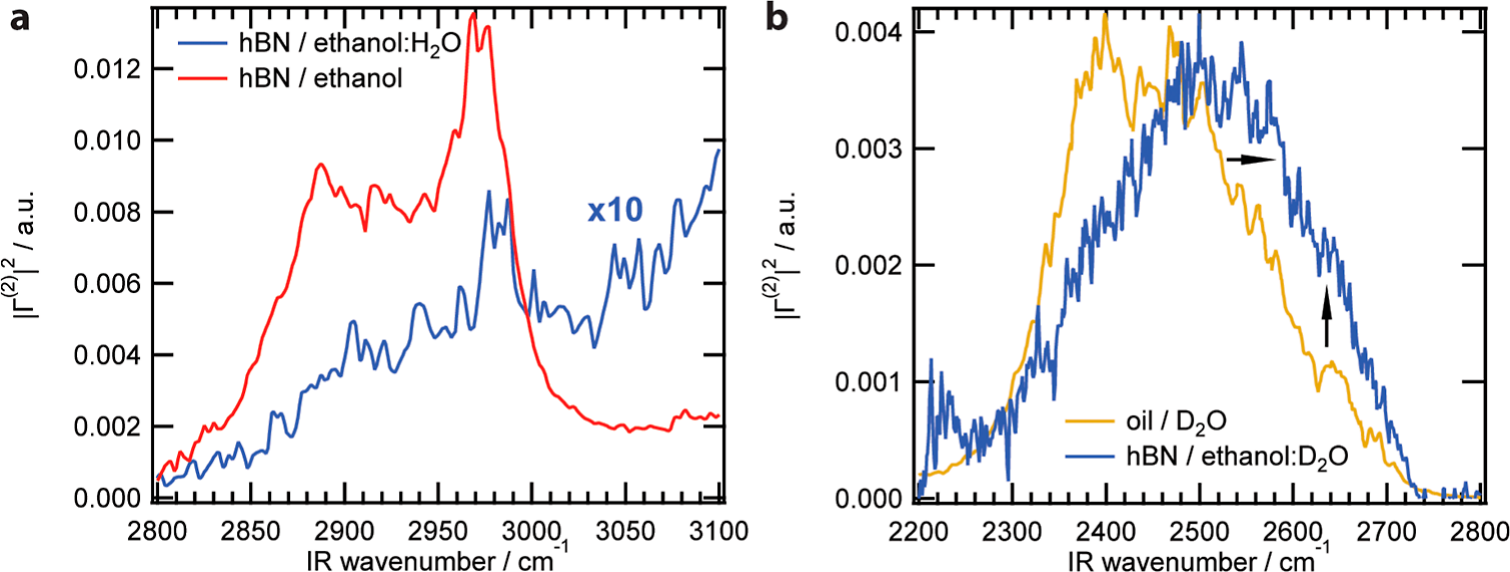
Solvent molecule - hBN interfacial structure. (a) SFS spectra in the C–H stretching region for hBN suspensions in neat ethanol and the ethanol/water mixture. (b) Comparison of SFS spectra of hBN suspensions in the ethanol-*d*_6_/D_2_O mixture to the oil-heavy water interface in the O–D stretching region.

### Probing Interfacial Water and Charge Transfer at the Nanoflake Surface

To investigate the presence of interfacial water, the SFS spectrum in the O–D stretching region was measured for hBN nanoflakes dispersed in an ethanol/water mixture (Figure 3b). In order to compare this data to earlier studies, heavy water mixed with ethanol-d_6_ was used as the main phase. The broad, largely featureless spectrum with a maximum around 2500 cm^–1^ is characteristic of interfacial water.^25,26^ The line shape is similar to water in contact with 100–200 nm radius hexadecane droplets (Figure 3b, yellow trace^26^), which suggests that the hBN interface has a hydrophobic character.^42^ The oil droplet water SFS spectrum consists of previously identified broad features that are also present at the air–water interface^25,43,44^ around 2395 cm^–1^ and 2500 cm^–1^ that correspond to H-bonded water molecules at the interface, with the 2395 cm^–1^ modes being more strongly H-bonded compared to the ones at 2500 cm^–1^. Approximately half of the spectral broadening arises from vibrational coupling.^45^ The shoulder at ∼2640 cm^–1^ was attributed to O–D bonds that are under-coordinated with other water molecules. Rather, these water molecules participate in weak improper H-bonds with the C–H groups of the oil. These improper H-bonds are responsible for the transfer of charge from water to oil^46^ (computed by ab initio MD simulations to be ∼0.015 electrons/nm^2^) which generates the negative charge on the oil droplets that is measured in electrophoretic mobility measurements. This surface charge imparts kinetic stability. The difference between the oil/water and hBN/water–ethanol interface is that the O–D spectrum has shifted to higher frequency. This means that the H-bond network at the hBN flake interface is weaker compared to the H-bond network at the oil nanodroplet water interface.

For oil nanodroplets suspended in water, charge transfer between O–H and C–H groups generates a net negative charge on the oil droplet. The droplets’ electrokinetic mobility depends on the H-bond network of water, which inherently enables the transfer of partial charges within and between H-bonds.^26,45^ The 3D H-bonding network enables the delocalization of partial positive charges around the nanodroplet. Applying an external electrostatic field causes the droplets to migrate toward the positive electrode, yielding a negative electrophoretic mobility. This role of the H-bond network is further emphasized at high pH, where the bulk pH modifies the conductivity of water to charge via the Grotthuss mechanism, increasing the mobility of the droplets. Optical second-harmonic scattering measurements of the surface potential of the oil nanodroplets in aqueous solution with identical ionic strengths but different pH revealed a pH-independent surface charge. Yet, mobility increased with pH, correlating exactly with enhanced charge conductivity. Therefore, it was concluded that the pH-dependent mobility of charged oil droplets in water depends on the resistance of charge motion through the H-bond network of water.^47^

### Connecting Molecular and Charge Related Information

Considering now the case of hBN nanoflakes dispersed in ethanol/water, Figure 3 shows that both water and ethanol are present at the interface. These liquids are highly miscible, and there is likely a three-dimensional H-bonded network formed between water and ethanol. The blue-shifted B–N vibrations, together with the 2640 cm^–1^ feature in the O–D spectrum are indicators of improper H-bond formation between B–N and O–D groups. These interactions create a negatively charged flake surface through electron donation from the O–D groups of water and ethanol to the flake. Due to this movement of partial charges (charge density displacement), which is possible within the H-bond network of the water/ethanol mixture, the liquid adjacent to the flake becomes slightly positively charged. As in the case of suspended oil droplets, these partial charges in the liquid phase are mobile and delocalized through the H-bond network. Under the influence of an external field, the hBN nanoflake will move toward the positive electrode, analogous to the behavior of the oil nanodroplets. This is illustrated in Figure 4a.

**Figure 4:**
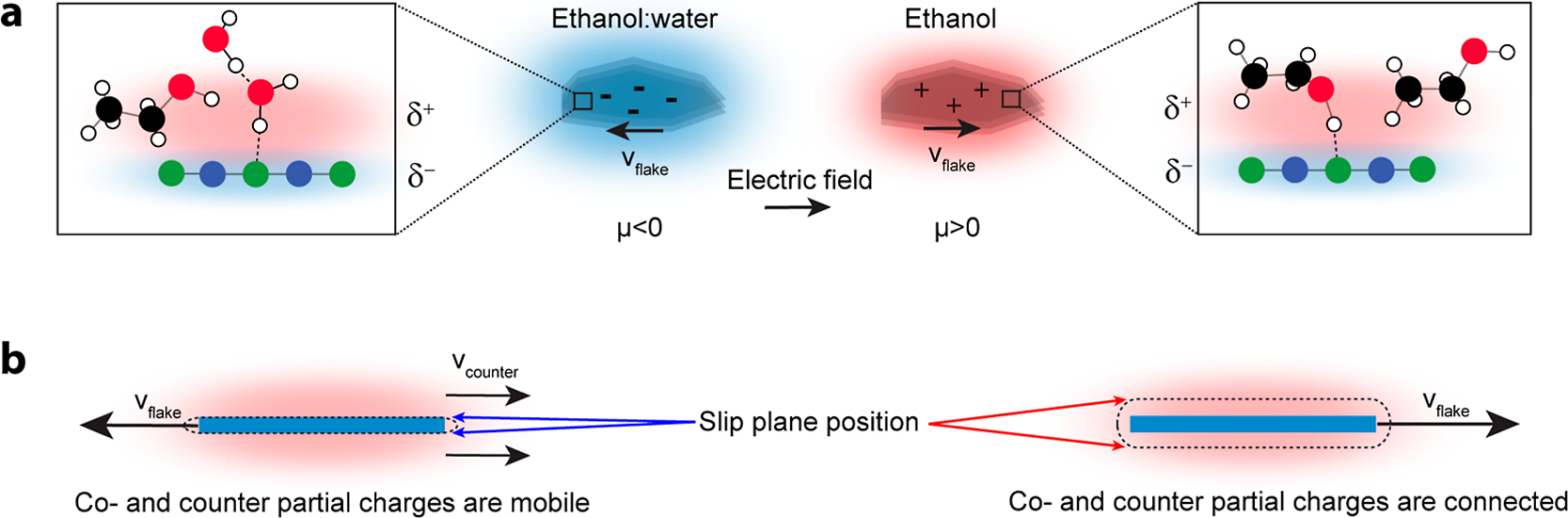
Molecular interpretation of the charging mechanism. (a) For both the ethanol/water mixture and neat ethanol, H-bonding with the hBN nanoflake occurs, resulting in charge transfer (δ_±_). In the presence of water (top-left), this charge transfer is delocalized through the three-dimensional H-bonding network. (b) As a result, hBN nanoflakes are electrophoretically driven without their counter-charge cloud in the presence of water, and with their counter-charge cloud in the absence of water, explaining the mobility change between solvent systems. The slip plane position is located close to the surface in the presence of water and further away in ethanol (dashed lines).

In the absence of water, charge transfer between the flake and ethanol can still occur via improper H-bonds. The B–N mode spectrum of nanoflakes in ethanol in Figure 2b (red line) is still blue-shifted compared to the dry flake spectrum, but less so than the spectrum of nanoflakes in the ethanol/water mixture. This suggests that there is still an overall net donation of electron density from ethanol molecules to the hBN flake. This net charge donation creates a partially negative flake surface in contact with partially positive ethanol molecules. Ethanol and water both exhibit H-bonding, but the extent over which this happens is notably different: water has a more connected three-dimensional H-bond network than ethanol owing to water's higher molar density and each water molecule participating in more H-bonds than each ethanol molecule, limiting the possibility of charge delocalization in ethanol. A charge separation will thus result in a flake-solvent ensemble that has a weakly positive charge localized on interfacial ethanol molecules, illustrated in Figure 4b. Therefore, when subjected to an external electrostatic field, the combined flake-ethanol object will move to the negative electrode. Thus, the apparent charge inversion presented in Figure 1 appears to be a manifestation of a different balance/spatial distribution of molecular-level charge transfer interactions. Another way of describing this phenomenon is in terms of the slip plane, which is the dividing plane between the surface-fixed charge and the freely moving liquid. For the ethanol/water mixture, it will be located between the partially negative surface charge and the mobile partially positive charge on the water molecules (Figure 4a). For the flakes in pure ethanol the slip plane is beyond the cloud of positive charge that is localized on the ethanol molecules. This means that the ‘flake charge’ as observed by ELS is a manifestation of charge distribution rather than magnitude/sign.

## Conclusions

In summary, although 2D nanoflakes made out of 2D materials have received a significant amount of attention due to their importance in nanofluidics, catalysis, and solution-based 2D material processing, up until now very little information is known about their molecular interfacial properties. In this work, we obtained molecular level understanding of the interactions between hexagonal boron nitride flakes and liquids. We find that hBN nanoflake charging behavior is intimately coupled to interfacial effects involving the solvent molecules. Notably, the ζ-potential determined by electrokinetic mobility measurements of pristine hBN nanoflakes exhibited an opposite sign when dispersed in neat ethanol and an ethanol/water mixture. Using SFS to probe the vibrational modes of the hBN/ethanol and hBN/ethanol–water interfaces, we observed the B–N spectra were significantly and progressively blue-shifted relative to that of a dry, drop-casted hBN film. This blue-shift was indicative of flake–liquid interactions. We observed C–H modes that verified the presence of interfacial ethanol under both solvent systems. In the presence of (heavy) water, we observed a reduction in C–H mode intensity as well as the presence of O–D modes, which indicated that water was in contact with the flake. Comparing the O–D spectrum of the hBN/ethanol–water interface to that of the oil nanodroplet/water interface showed a similarity between the water structures. In particular, the spectral feature that is identified as a charge transfer mode was present in both systems, suggesting that there are improper H-bonds between the flake and the O–H groups of water or ethanol. These bonds facilitate charge transfer of electron density that renders the B–N surface of the flake slightly negative and the interfacial liquid slightly positive for both ethanol and the ethanol–water mixture. In the ethanol/water mixture the partial positive charge is mobile owing to the 3D H-bond network of water, resulting in a negative electrophoretic mobility, as the negatively charged flake can move independent from the positive charge in the solution. The partially positive charge adjacent to the flake in the pure ethanol phase is more immobile, resulting in a positive electrophoretic mobility. This interpretation is consistent with the direction of the spectral shift observed in the B–N modes of suspended hBN nanoflakes relative to the dry film, with the larger blue-shift in the presence of water due to enhanced charge transfer. Examining the interfacial structure thus reveals an array of molecular-level interactions that are crucial for understanding the properties of hBN nanoflakes and their broader implications for 2D materials. These insights advance our knowledge of hBN-liquid interactions, which are critical for the development of liquid-exfoliated 2D material inks.

## Materials and Methods

### Materials

Pristine boron nitride (BN) flakes in solution were obtained from Graphene Supermarket, and had the following parameters: lateral size: 50–200 nm, thickness: 1–5 monolayers, purity in dry phase ≥99%. Dispersions of 5.4 mg/L BN in 55 v/v % ethanol/water were used to prepare hBN solutions in ethanol, ethanol-*d*_6_, and 55 v/v % ethanol-*d*_6_/D_2_O. Ethanol (≥99.5%, Sigma-Aldrich), heavy water (D_2_O) (99.9 atom % D, Sigma-Aldrich) and deuterated ethanol (ethanol-d_6_ anhydrous, ≥99.5 atom %D, Sigma-Aldrich) were used as received. Thorough characterization of the flakes used was performed by Krečmarová et al.,^32^ using μ-Raman spectroscopy, scanning electron microscopy and transmission electron microscopy by Khan et al.^33^ Solutions were transferred to cuvettes consisting of a CaF_2_ front window and a quartz back window containing a 200 μm channel for SFS measurements or a 10 μm channel for IR transmittance measurements. Sulfuric acid and hydrogen peroxide were used in a 3:1 ratio for piranha cleaning. 70 v/v % ethanol/water and ultrapure water were used for sample rinsing and sonication. All materials were used without further purification.

### Sample Preparation

Boron nitride pristine flakes in 55 v/v % ethanol/water (determined from the density^48^) were sonicated (30 min) and then cooled in an ice–water bath (30 min). The chilled solution was centrifuged (1 h, 16,000 RCF) and the supernatant was decanted. The supernatant was measured by UV–vis spectroscopy to determine successful solvent exchange (Figure S3). The BN pellets were placed under vacuum (1 h) before redispersion in desired solvent. The redispersed BN flakes were sonicated (0–30 °C, 30 min per cycle) and cooled in an ice bath repeatedly before equilibrating in a room temperature water bath (10 min) prior to SFS measurement. Flake size and concentration were measured by dynamic light scattering (DLS). The CaF_2_ and quartz cuvette windows were sonicated (20–70 °C, 30 min) in 70 v/v % EtOH/H_2_O and rinsed with ultrapure water prior to submersion in piranha solution. The quartz window was submerged for a few minutes while the CaF_2_ window was submerged and removed immediately to prevent significant dissolution. The windows were copiously rinsed with ultrapure water and then stored in ultrapure water until SFS measurement. Prior to use, the cuvette windows were rinsed with 70 v/v % EtOH/H_2_O and ultrapure water before drying with compressed air.

### IR Measurements

One mL of the stock solution was drop-casted on the ZnSe crystal of a commercial ATR-IR apparatus (Bruker Vertex 70 FTIR spectrometer). The deposit was pressed against the crystal using the metallic arm and the mid-infrared beam directed toward the sample at 45° to achieve total internal reflection. The spectrum shown in Figure 2c corresponds to a measurement of the deposit, corrected for the background measured without the deposit. Infrared transmittances were measured using FTIR spectroscopy. Transmittance spectra were recorded in a CaF_2_/quartz cuvette containing a 10 μm channel and again in a CaF_2_/CaF_2_ cuvette with a solution of unknown thickness. The latter sample configuration was required to measure the full transmittance spectrum in both the C–H stretching (∼2700–3300 cm^–1^) and C–H bending (∼1300–1600 cm^–1^) regions, while the former sample configuration was required to scale the full spectrum to a known thickness of 10 μm using the Beer–Lambert law. Infrared transmittances were also measured using the ultrafast mid-IR laser generated in the TOPAS-NDFG by introducing the same sample cuvettes described above into the beam path at an incident angle of 45° and measuring the nonresonant spectrum of the BaTiO_3_ nanoparticle film.

### Raman Scattering

100 μL of the stock solution was drop-casted on a silicon wafer and imaged using a commercial scanning point microscope equipped with a spectrometer (Renishaw). The sample was illuminated with a 532 nm laser focused to a ∼1 μm^2^ point. Scattered light was collected using a 100× air objective, and dispersed with a grating of 3000 gr/mm. A map of 10 × 10 μm of the deposit was imaged by scanning the sample, and the spectrum presented in Figure 2c corresponds to the average over the many nanoflakes found in the scanned region.

### Dynamic Light Scattering and Electrophoretic Light Scattering Measurements

The average size, polydispersity, and particle concentration were measured using dynamic light scattering (ZetaSizer, Malvern). The ζ-potential was calculated from measured electrophoretic mobilities according to $\mu =\frac{\epsilon_{0}\epsilon \zeta f\left(\kappa R\right)}{\eta }$ , where μ is the electrophoretic mobility, ϵ_0_ is the vacuum permittivity, ϵ is the relative permittivity of water, *k* is the inverse Debye length, *R* is the radius, η is the viscosity, and *f*(κ*R*) is the Henry function (Smoluchowski approximation, *f* = 1). Particle concentrations were calculated from a three-scattering angle measurement and the MADLS algorithm. Particle concentrations were determined to be on the order of 10^8^ ± 10^7^ particles/mL. Average sizes varied between 100 and 250 nm for the experimental replicates of hBN flakes dispersed in the different solvent systems, while polydispersity varied from 0.1 to 0.3. No dilution was needed for these measurements.

### Laser Assembly

Details of the laser assembly can be found elsewhere.^49^ Briefly, a regeneratively amplified laser (Spectra-Physics, Spitfire Pro, 1 kHz, 100 fs, 7 W) was seeded by a Ti-sapphire oscillator (Spectra-Physics, Mai Tai, 80 MHz, 0.83 W) and two Nd/YLF lasers (Spectra-Physics, Empower 30) to generate ultrafast, high peak power 800 nm pulses. Two thirds of this amplified 800 nm beam was directed into a noncollinear optical parametric amplifier (Light Conversion, HE-TOPAS-C/NDFG) to generate tunable, broadband mid-IR pulses (fwhm = ∼100 cm^–1^). The mid-IR light was passed through a filter to remove residual signal and idler and the polarization was controlled by a pair of BaF_2_ wire grid polarizers before being focused on the sample by an uncoated off-axis parabolic gold mirror at a 45° angle incident to the sample normal with a pulse energy of 10 μJ. The IR beam path was purged with nitrogen gas (4 bar). The remaining one-third of the amplified 800 nm beam was passed through a homemade pulse-shaper to expand and stretch the pulse in time and frequency, respectively, to produce narrow (fwhm = ∼10 cm^–1^) pulses of picosecond duration. These picosecond pulses were passed through a delay stage, a pair of polarizers, and a half-wave plate before being focused onto the sample with a pulse energy of 10 μJ. The generated sum frequency light was collected by a lens normal to the sample (55° in air relative to the forward scattering direction). The sum frequency light was passed through a half-wave plate, a polarizer, two filters, prior to focusing into a spectrometer by a lens. The light was detected on an intensified CCD camera.

### Sum Frequency Scattering

A CaF_2_/quartz cuvette containing a thin film of BaTiO_3_ nanoparticles was mounted to the sample stage and measured by SFS using IR frequencies centered around ∼1385 cm^–1^, ∼2160 cm^–1^, and ∼2960 cm^–1^ to measure the spectral line shape of the laser pulses using the nonresonant BaTiO_3_ response. The BaTiO_3_ sample was exchanged with a clean cuvette containing solvent without flakes and measured as background. The solvent was then exchanged for freshly prepared hBN flakes in solution and measured. The background spectra were subtracted from the sample spectra with flakes and then divided by the BaTiO_3_ reference spectra. Background spectra were always measured prior to suspensions to avoid contamination of the cuvette with nanoflakes. All spectra were collected in the SSP (S-sum frequency, S-visible, P-infrared) polarization. The detected frequency was calibrated by referencing the known absorption bands of a polystyrene film introduced into the IR beam path. SFS spectra were further processed to account for the IR absorbance through the sample medium as described previously (see Supporting Information).^26,37,38^

## Supplementary Material



## Notes

Size distributions measured by DLS, FTIR spectra of solvent systems, UV–vis absorbance spectra of samples during preparation, SFS collection function, SFS correction factor (PDF)
