# Supplementary material for: Sum-Frequency Scattering Spectroscopy Reveals the Charging Mechanism and Surface Structure of hBN Nanoflakes in Solution
Source: ACS Nano. 2025 Jul 3;19(27):24912–9. doi: 10.1021/acsnano.5c03589 (PMC12269351; doi:10.1021/acsnano.5c03589)
Supplement: Supplementary file 1 [file nn5c03589_si_001.pdf]

Supporting Information for

Sum-frequency scattering spectroscopy reveals the charging mechanism and surface structure of hBN nanoflakes in solution

Benjamin Rehl,[†,¶] Nathan Ronceray,[‡,¶] Li Zhang,[†] Aleksandra Radenovic,[‡] and Sylvie Roke[*,†]

†Laboratory for fundamental BioPhotonics, Institute of Bioengineering (IBI), School of Engineering (STI), École Polytechnique Fédérale de Lausanne (EPFL), CH-1015 Lausanne, Switzerland.; Institute of Materials Science and Engineering (IMX), School of Engineering (STI), École Polytechnique Fédérale de Lausanne (EPFL), CH-1015 Lausanne, Switzerland.; Lausanne Centre for Ultrafast Science, École Polytechnique Fédérale de Lausanne (EPFL), CH-1015 Lausanne, Switzerland.

‡Laboratory of Nanoscale Biology (LBEN), Institute of Bioengineering, School of Engineering, Swiss Federal Institute of Technology Lausanne (EPFL), 1015 Lausanne, Switzerland; NCCR Bio-Inspired Materials, École Polytechnique Fédérale de Lausanne, 1015 Lausanne, Switzerland

¶*Equal contribution*

E-mail: sylvie.roke@epfl.ch

Contents

Figure S1. Size distributions measured by DLS .... page 2

Figure S2. FTIR spectra of solvent systems .... page 3

Figure S3. UV-Vis absorbance spectra of samples during preparation .... page 4

Figure S4. SFS collection function .... page 5

Figure S5. SFS correction factor .... page 6

References .... page 7

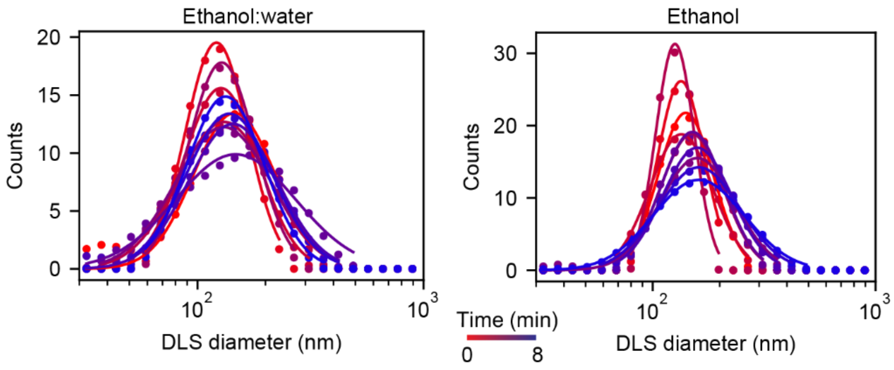

Figure S1. Size distributions measured by DLS. For each solvent system (ethanol:water on the left, ethanol on the right) one measurement is acquired each minute for 8 minutes, showing a relative stability of the flakes with a broadening of the distribution that is attributed to a slow aggregation process. Fits correspond to log-normal distributions used to obtain the average and standard deviation reported in Fig. 1a.

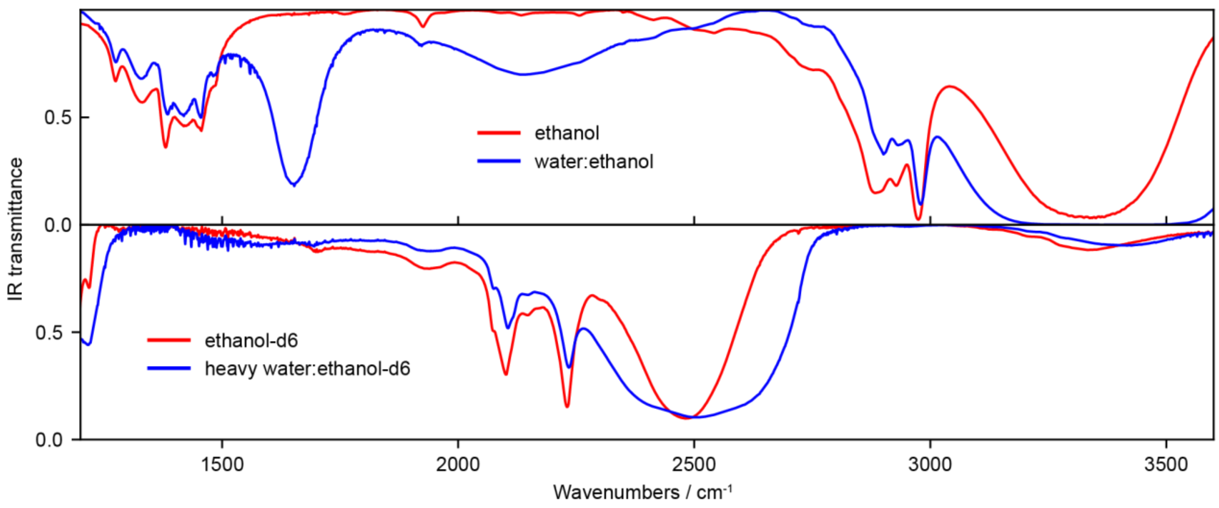

Figure S2. FTIR spectra of the solvents. Transmittance spectra of the protonated (top) and deuterated (bottom) solvent systems equivalent to a 10 um channel between CaF_2 windows.

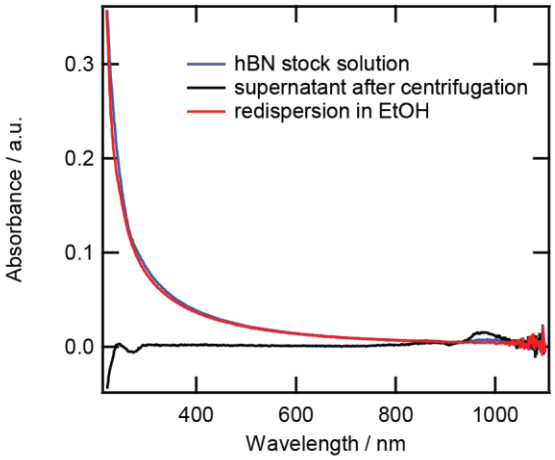

Figure S3. **UV-Vis absorbance spectra of samples during preparation.** UV-Vis absorbance of stock solution (hBN in 55 v/v% EtOH:H_2O), supernatant following centrifugation, and redispersed flakes in ethanol. The lack of absorbance in the supernatants indicates minimal material was lost during solvent exchange.

SFS collection function. The effective particle susceptibility, $\Gamma^{(2)}(\omega_{IR}, \omega_{vis})$, is related to the experimentally measured sum frequency intensity, I_{SF}, by

$$\left|\Gamma^{(2)}(\omega_{IR}, \omega_{vis})\right|^2 \propto \frac{I_{SF}(\omega_{IR}, \omega_{vis})}{\int_0^L I_{IR}(\omega_{IR}) I_{vis}(\omega_{vis}) f_{focal}(z)\rho(z)dz}, \quad \text{(eq. S1)}$$

where I_{IR} is the intensity of IR light, I_{vis} is the intensity of visible light, f_{focal} is the collection function, $\rho(z)$ is the particle distribution, and z and L are the optical axis and sample length, respectively. The product of the collection function and particle distribution, $f_{focal}(z)\rho(z)$, can be determined by measuring the SF intensity of the sample off-resonance and where IR absorption is minimal (Figure S4). We estimated this product as Gaussian.

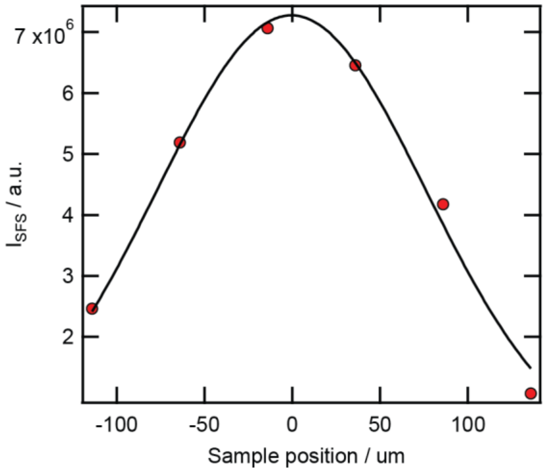

Figure S4. SFS collection function. The lens collection profile and particle distribution of hBN flakes in solution was measured by scanning the sample along the z-axis (IR beam direction), integrating the non-resonant SFS response (~2550 cm^{-1} for protonated samples) of hBN flakes dispersed in solvent, and fitting to a Gaussian.

SFS correction factor. Since the medium absorbs IR light as it transits the sample, I_{IR} is described by the Beer-Lambert law as $I_{IR} = I_{IR,0}(\omega_{IR})e^{-\alpha(z)z}$, where $I_{IR,0}$ is the initial IR light intensity and $\alpha(z)$ is the absorbance. We can therefore rearrange eq. S1 to the following

$$\left|\Gamma^{(2)}(\omega_{IR},\omega_{vis})\right|^2 \propto \frac{I_{SF}(\omega_{IR},\omega_{vis})}{I_{IR,0}(\omega_{IR})I_{vis}(\omega_{vis})\int_0^L e^{-\alpha(z)z} f_{focal}(z)\rho(z)dz} = \frac{I_{SF}(\omega_{IR},\omega_{vis})}{I_{IR,0}(\omega_{IR})I_{vis}(\omega_{vis})c(\omega_{IR})}. \quad \text{(eq. S2)}$$

The coupled lens collection profile and particle distribution product and exponential decay of the IR intensity due to solvent absorption is integrated over the sample length to determine the correction factor, $c(\omega_{IR})$ (Figure S5). $I_{IR,0}$ and I_{vis} are determined by measuring the nonresonant intensity of a reference material (ie. a $BaTiO_3$ film) as described in the methods.

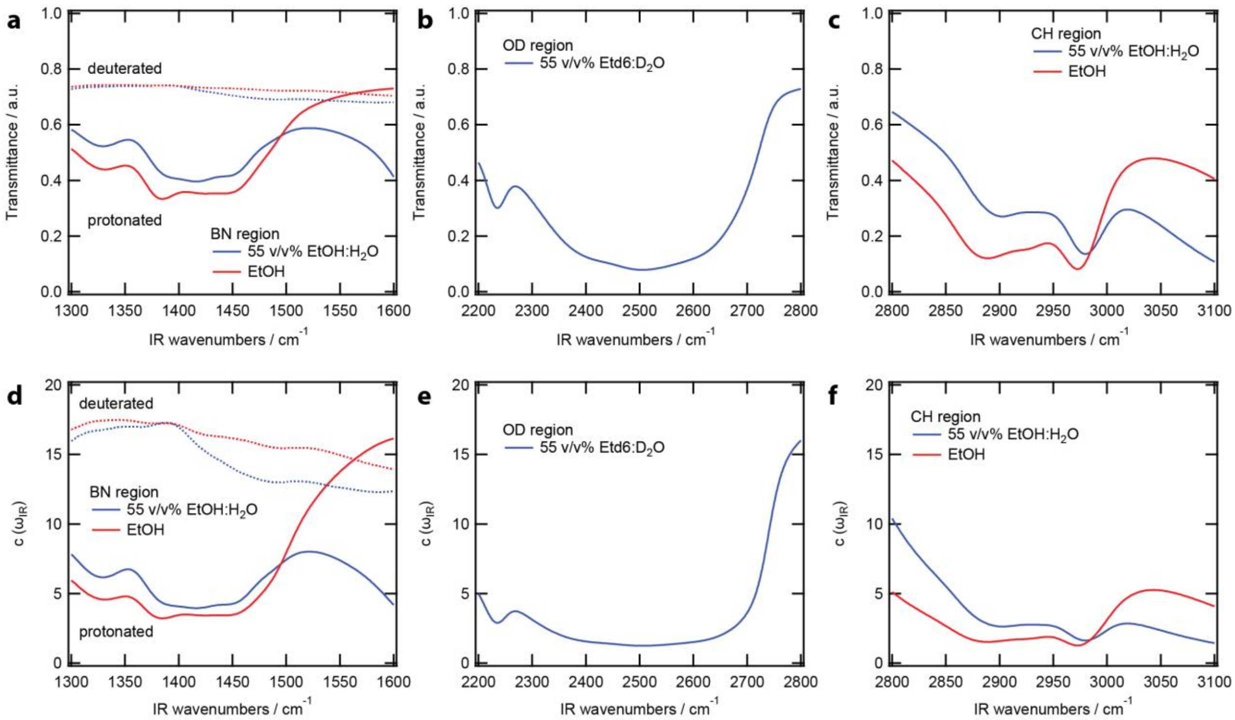

Figure S5. SFS IR absorbance correction profiles. The IR transmittances measured by FTIR were scaled and smoothed using a Gaussian smoothing procedure to account for experimental differences in spectral resolution between FTIR and SFS (a-c). The modified IR transmittance described by the Beer-Lambert law coupled with the lens collection profile and particle distribution was integrated over the path length of the IR beam through the sample (d-f). The SFS spectra were then normalized by this integral to provide the final $|\Gamma^{(2)}|^2$ spectrum.

References

1. Krečmarová, M.; Canet-Albiach, R.; Pashaei-Adl, H.; Gorji, S.; Muñoz-Matutano, G.; Nesládek, M.; Martínez-Pastor, J. P.; Sánchez-Royo, J. F., Extrinsic Effects on the Optical Properties of Surface Color Defects Generated in Hexagonal Boron Nitride Nanosheets. *ACS Applied Materials & Interfaces* **2021,** *13* (38), 46105-46116.
2. Khan, A. F.; Randviir, E. P.; Brownson, D. A. C.; Ji, X.; Smith, G. C.; Banks, C. E., 2D Hexagonal Boron Nitride (2D-hBN) Explored as a Potential Electrocatalyst for the Oxygen Reduction Reaction. *Electroanalysis* **2017,** *29* (2), 622-634.
3. Khattab, I. S.; Bandarkar, F.; Fakhree, M. A. A.; Jouyban, A., Density, viscosity, and surface tension of water+ethanol mixtures from 293 to 323K. *Korean Journal of Chemical Engineering* **2012,** *29* (6), 812-817.
4. de Aguiar, H. B.; Samson, J.-S.; Roke, S., Probing nanoscopic droplet interfaces in aqueous solution with vibrational sum-frequency scattering: A study of the effects of path length, droplet density and pulse energy. *Chemical Physics Letters* **2011,** *512* (1), 76-80.
5. Pullanchery, S.; Kulik, S.; Rehl, B.; Hassanali, A.; Roke, S., Charge transfer across C–H···O hydrogen bonds stabilizes oil droplets in water. *Science* **2021,** *374* (6573), 1366-1370.
6. Kulik, S.; Pullanchery, S.; Roke, S., Vibrational Sum Frequency Scattering in Absorptive Media: A Theoretical Case Study of Nano-objects in Water. *The Journal of Physical Chemistry C* **2020,** *124* (42), 23078-23085.
7. Pullanchery, S.; Zhang, L.; Kulik, S.; Roke, S., Interfacial Inversion, Interference, and IR Absorption in Vibrational Sum Frequency Scattering Experiments. *The Journal of Physical Chemistry B* **2023,** *127* (30), 6795-6803.
